# The MAP2K5-linked SNP rs2241423 is associated with BMI and obesity in two cohorts of Swedish and Greek children

**DOI:** 10.1186/1471-2350-13-36

**Published:** 2012-05-17

**Authors:** Mathias Rask-Andersen, Josefin A Jacobsson, George Moschonis, Anna E Ek, George P Chrousos, Claude Marcus, Yannis Manios, Robert Fredriksson, Helgi B Schiöth

**Affiliations:** 1 Department of Neuroscience, Functional Pharmacology, Uppsala University, BMC, Uppsala SE 75124, Sweden; 2 Department of Nutrition & Dietetics, Harokopio University of Athens, Athens, Greece; 3 Department for Clinical Science, Intervention and Technology (CLINTEC), department of Pediatrics, Karolinska Institute, Karolinska University Hospital, Huddinge, B57, 141 86, Stockholm, Sweden; 4 First Department of Pediatrics, Athens University Medical School, Aghia Sophia Children’s Hospital, Thivon and Levadias, 115 27, Goudi, Athens, Greece; 5 Pediatrics, National Childhood Obesity Centre, Stockholm, Sweden

**Keywords:** Obesity, MAP2K5, Childhood obesity, Genetics, rs2241423

## Abstract

**Background:**

Recent genome-wide association studies have identified a single nucleotide polymorphism within the last intron of MAP2K5 associated with a higher body mass index (BMI) in adults. MAP2K5 is a component of the MAPK-family intracellular signaling pathways, responding to extracellular growth factors such as brain derived neurotrophic factor (BDNF) and nerve growth factor (NGF). In this study, we examined the association of this variant in two cohorts of children from Sweden and Greece.

**Methods:**

We examine the association of rs2241423 to BMI in a cohort of 474 Swedish children admitted for treatment of childhood obesity and 519 children matched for gender, ethnicity and socioeconomic background from the Stockholm area, as well as a cross-sectional cohort of 2308 Greek school children (Healthy Growth Study). Children were genotyped using a predesigned TaqMan polymorphism assay. Logistic regression was used to test for an association of rs2241423 to obesity in the cohort of Swedish children. Linear regression was used to test for an association of rs2241423 to BMI z-score and phenotypic measurements of body adiposity in the cohort of Greek children. Models were adjusted for age and gender. In the cohort of Greek children the model was also adjusted for stage of pubertal development.

**Results:**

The minor allele of rs2241423, allele A, was associated with a protective effect against obesity in the cohort of Swedish children (*p* = 0.029, OR = 0.79 (95% CI: 0.64–0.98)), and with a lower BMI z-score in the cohort of Greek children (*p* = 0.028, *β* = −0.092). No association to phenotypic measurements of body fat distribution could be observed in our study.

**Conclusions:**

rs2241423 was associated with BMI and obesity in two independent European cohorts suggesting a role for MAP2K5 in early weight regulation.

## Background

**Figure 1:**
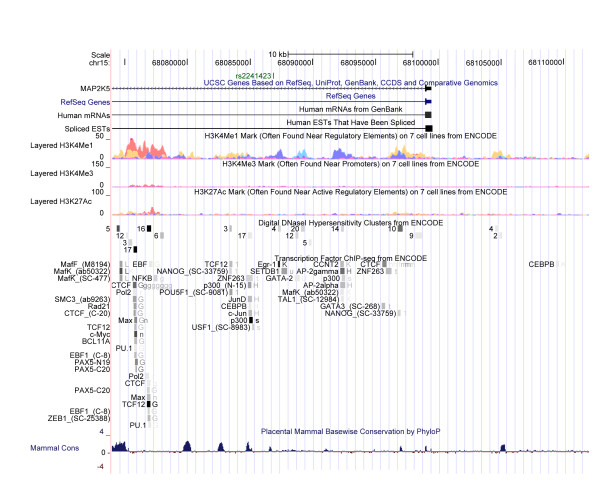
**Visualization of DNA elements in the rs2241423-associated haplotype block utilizing ENCODE.** The haplotype block containing rs2214123 was visualized employing ENCODE (ENCyclopedia Of DNA Elements) data tracks via the UCSC genome browser (http://genome.ucsc.edu/). We chose to visualize regulatory binding sites generated from transcription factor chromatin-immunoprecipitation assays followed by sequencing (ChIP-seq), histone modifications, DNaseI hypersensitivity clusters, as well as mammalian conservation. Rs2214123 was found to be located in close proximity to a number of potential regulatory sites. The closest cluster was found only 400 bases downstream of rs2241423. Among the DNA-interacting transcription factors identified as binding to this site, early growth response protein 1 (EGR1) gave the strongest signal for occupancy. In addition, this site also overlaps with a signal peak for monomethylation of lysine 4 on histone H3 (H3K4Me1), as identified by ChIP-seq in K526 cells, a human erythroleukemia cell line, as well as DNAseI activity which indicates chromatin modifications consistent with a regulatory element at this position. An additional cluster of regulatory elements was identified approximately 10 kb upstream of rs2241423 exhibiting strong signals for H3K4Me1 in several cell lines as well as for lysine 27 acetylated histone H3, an indicator of active regulatory elements. This site also overlaps with strong signals for DNaseI hypersensitivity clusters which are characteristic of cis-regulatory elements. DNA from this cluster was found to co-precipitate with antibodies for several different transcription factors, strong signals for occupancy in this cluster were observed for transcription factor 12 (TCF12) and cMyc.

A strong genetic component has been shown to underlie the development of common polygenic obesity, one of the most serious medical problems facing industrialized societies today. Over the last decade, large scale genome wide association studies (GWAs) have identified some of the genetic variants associated with obesity. A recent report from the GIANT-consortium, a meta-analysis of GWAs on a total of nearly 250000 individuals, identified 32 loci associated with an increased risk of developing obesity [1]. The GIANT consortium also reported directionally consistent effects of rs2241423 in meta-analyses of the cohorts containing children included in the main analysis [1]. However, a statistically significant effect was observed in the meta-analysis of extremely obese case–control association studies (n = 15 250) and transmission disequilibrium tests (TDT) (n = 1 460), but not in the meta-analysis of population-based studies (n = 1 460) [1]. Variations in the gene encoding the dual specificity mitogen-activated protein kinase kinase 5 (MAP2K5) were subsequently identified as risk factors for obesity in a meta-analysis of data from five cohorts of Chinese, Malay and Indian descent (total n = 10 482) [2]. In this study, the strongest association to BMI was identified as the major allele of the single nucleotide polymorphism (SNP) rs2241423 located in the last intron of the MAP2K5 gene and in proximity of the gene encoding SKI family transcriptional corepressor 1 (SKOR1) (Figure 1, Additional file 1: Figure S1).

This locus has previously been associated with the restless legs syndrome (RLS) [3], a condition defined by an urge to move the legs often accompanied by uncomfortable or unpleasant sensations in the legs temporarily relieved during movement. The symptoms of RLS often worsen during periods of rest and can cause difficulties falling asleep as well as sleep disruptions which can seriously affect quality of life [4]. Common hereditary factors between RLS and obesity have also previously been suggested [5].

MAP2K5 has been extensively studied as it is a component of the MAPK-family intracellular signaling pathways, responding to extracellular growth factors such as brain derived neurotrophic factor (BDNF), nerve growth factor (NGF) (Kyoto Encyclopedia of Genes and Genomes, KEGG [6]), insulin-like growth factor 2 (IGF2) [7], granulocyte colony-stimulating factor [8] as well as epidermal growth factor [9]. This pathway also responds to shear stress [10] and osmotic stress. MAP2K5 is a 20 exon gene spanning about 280 k bp. It encodes three known protein-coding transcripts, one of which MEKα, is expressed in the liver and the brain to a higher degree [11] where it acts to phosphorylate and activate extracellular signal regulated kinase 5 (ERK5) which in turn activates transcription factors, also via phosphorylation, leading to cell differentiation and proliferation [12]. MEKβ on the other hand is ubiquitously expressed but does not activate ERK5. MEKβ has instead been suggested to act as an inhibitor of ERK5-activation via competitive binding, and to inhibit cell growth in terminally differentiated cell types.

Rs2241423 is furthermore located 30 kb upstream of the gene encoding SKOR1. SKOR1 is a transcriptional co-repressor which acts by inhibiting gene expression induced by bone morphogenetic (BMP) family growth factors [13,14]. In humans, SKOR1 is mainly expressed in the adult cerebellum [14], but it may play a more important role as an induced repressor of gene transcription and subsequent cellular differentiation during early central nervous system (CNS) development. Mouse studies have shown this gene to be important for determining cell fate in the developing CNS [14,15]. SKOR1 is also involved in wound healing [13].

In this study we aim to further explore the association of the MAP2K5-linked SNP, rs2241423, to obesity and BMI in two cohorts of Swedish and Greek children. The high allele frequency of rs2241423, and the effects observed in meta-analysis of child cohorts by the GIANT consortium, makes it a good candidate for association studies in our cohorts. We also tested for an association to phenotypic variables describing body fat distribution in the cohort of Greek children, with the aim to specify the effect carried by the genetic variant. Identification and replication of genetic factors underlying pediatric obesity is also of great interest as genetic variants can have differential effects on early-onset and adult obesity. Rs2241423 was genotyped in two cohorts: one consisting of 474 children admitted to the pediatric clinic for treatment of childhood obesity at the National Childhood Obesity Centre, Karolinska University Hospital, Sweden, as well as an age- and gender-matched control population of 519 children from the same area; and one cohort of 2308 children from primary schools in the Athens-region, Greece. We also tested the association of rs2241423 to phenotypic measurements of body-fat distribution in the cohort of Greek children.

## Methods

### Children from the Stockholm area of Sweden

Genotyping of rs2241423 was performed on 993 children and adolescents comprising two study groups as described earlier [16,17]. Briefly, one group of 474 obese children and adolescents (250 girls and 224 boys, age 6–21 years) enrolled at National Childhood Obesity Centre at the Karolinska University Hospital, Huddinge, Sweden and one group of 519 normal weight Swedish adolescents (267 girls and 252 boys, age 15–20 years) recruited from 17 upper secondary schools around Stockholm and matched to the obese group with respect to ethnicity and socioeconomic status (Table 1). Subjects with overweight/obesity or chronic diseases were excluded from the group of normal weight adolescents, and subjects with type 2 diabetes and known related subjects were excluded from the obese group. BMI was calculated from height and weight and body mass index standard deviation score (BMISDS) was calculated from weight and height and standardized for age and gender. Subjects with overweight/obesity or chronic diseases were excluded from the group of normal weight adolescents, and subjects with type 2 diabetes were excluded from the obese group. The study was approved by the Regional Committee of Ethics, Stockholm. Informed consent was provided by all participants or by their legal guardians.

**Table 1:** Descriptive characteristics of the Swedish and Greek child cohorts

***Greek children***	**All**	**Underweight**	**Normal Weight**	**Overweight**	**Obese**	**1-Way ANOVA**
N (boys/girls)	2308 (1163/1145)	69 (27/42)	1267 (617/650)	693 (354/339)	263 (157/106)	
Age (years)	11.2 ± 0.7	11.2 ± 0.7	11.2 ± 0.7	11.1 ± 0.7	11.1 ± 0.6	ns
Body weight (kg)	45.3 ± 11.2	29.6 ± 4.3	39.1 ± 6.0	50.7 ± 6.6	64.7 ± 9.7	***
Length (cm)	148 ± 7.9	144 ± 8.0	147 ± 7.8	150 ± 7.6	152 ± 7.5	***
BMI z-score	0.85 ± 1.23	−1.89 ± 0.47	0.09 ± 0.71	1.76 ± 0.34	2.81 ± 0.36	***
***Swedish children and adolescents***	**Control population**	**Obese Patients**				**t-test**
N (boys/girls)	519 (252/267)	474 (224/250)				
Age (years)	17.0 ± 0.9	12.8 ± 3.2				***
Body weight(kg)	63.5 ± 10.0	92.9 ± 29.1				***
Length (cm)	172.9 ± 8.9	159.1 ± 16.0				***
BMI z-score	0.08 ± 0.83	3.54 ± 0.60				***

Body mass index z-scores (BMI z-score) were calculated as described by Cole et al [18] using LMS variables from the international obesity task force (IOTF). Two-way analysis of variance (ANOVA) was used to compare between groups. Group means were compared to the normal weight group in the greek cohort and with the control population in the swedish cohort. *** - *p* < 0.0001. ns – not significant.

### Greek child cohort - the ‘healthy growth study’

The cohort of Greek children was comprised of 2658 schoolchildren, attending the 5th and 6th grades of primary schools (Table 1) and participating in the “Healthy Growth Study”, a large scale, cross-sectional and epidemiological study initiated in May 2007 as described previously [19,20]. An extended letter explaining the aims of the current study and a consent form were provided to each parent who had a child in one of the primary schools participating in the study. Those parents who agreed to participate in the study gave their informed consent by signing the consent form, and provided their contact details. Body weight and height were measured in all study participants using standard procedures and equipment. Body weight was measured to the nearest 10 g and height was measured to the nearest 0.1 cm in standing position. BMI z-score was calculated relative to the International Obesity Task Force (IOTF) definitions [21]. Waist and hip circumference was measured to the nearest 0.1 cm with the use of a non-elastic tape (Hoechstmass, Sulzback, Germany). Measurements were taken with the subject at a standing position, around the trunk, at the level of umbilicus midway between the lower rib margin and the iliac crest. The thickness of four skinfolds (triceps, biceps, subscapular and suprailiac) was measured to the nearest 0.1 mm to the right side of the body with a Lange skinfold caliper (Cambridge, Maryland). Each skinfold was grasped gently, in order to avoid causing any unnecessary discomfort to the child. Triceps and biceps skinfold thickness was measured with the right arm hanging relaxed at the side of the body while the skinfold was picked up about 1 cm bellow the midpoint mark over the triceps and biceps muscle respectively. Measurement of the subscapular skinfold thickness was performed while the child stood with shoulders relaxed and after identifying the inferior angle of the scapula. The skinfold was picked up 1 cm below the subscapular mark. Suprailiac skinfold was measured just above the iliac crest, along the axis of the anterior line. In each case the caliper was applied to the “neck” of the fold just above the finger and thumb, for two repeated measurements. Four well-trained and experienced female pediatricians determined pubertal maturation (Tanner Stage) after a thorough inspection [22]. Breast development in girls and genital development in boys was used for pubertal maturation classification (Tanner stages 1 to 5). DNA for genotyping was available for 2308 individuals (1163 males and 1145 females). Approval to the study was granted by the Greek ministry of Education and the Ethical Committee of Harokopio University of Athens. Informed consent was provided by all participants or by their legal guardians.

### Genotyping and linkage disequilibrium analysis

Genotyping of rs2241423 was carried out with a pre-designed Taqman single-nucleotide polymorphism genotyping assay (Applied Biosystems, Foster City, USA) and an ABI7900 genetic analyzer with SDS 2.2 software at the Uppsala Genome Center (http://www.genpat.uu.se/node462). The genotype call rate was 97.8% in the Swedish cohort and 99.0% in the Greek cohort. Tests for deviation from Hardy-Weinberg equilibrium were performed using a Markov chain Monte Carlo test [23] in PLINK [24]. No deviations from Hardy-Weinberg equilibrium were detected for rs2241423 in the Swedish and Greek cohorts.

### Linkage disequilibrium-analysis

Haploview [25] was used to generate a graphical representation of the linkage disequilibrium (LD) structure from r^2^ scores. The LD pattern was generated using HapMap data version 3, release 2 and CEU + TSI as analysis panel (Additional file 1: Figure S1).

### Statistical analyses

Association with obesity was analyzed using logistic regression in the Swedish cohort comparing children diagnosed as obese at the Huddinge Childhood Obesity Centre to normal weight controls recruited from the same region. Association was presented as an odds ratio (OR) with 95% confidence interval (CI) (Table 2). Gender was accounted for in the analysis. Linear regression analysis was used to ascertain association between rs2241423 and BMI and body adiposity measurements in the Greek cohort. Skewed quantitative variables were normalized by logarithmic transformation before analysis. Models were adjusted for age, gender, BMI and stage of pubertal development: tanner-stage [26]. Statistical analyses were performed with PLINK (http://pngu.mgh.harvard.edu/purcell/plink/) [24] assuming an additive model. Given the prior information about the role of *MAP2K5* variation in obesity, the evaluation of the association with BMI and obesity is considered a replication study; thus, nominal *p* values ≤ 0.05 were considered significant. For obesity related phenotypes in the Greek cohort not previously studied, we applied a Bonferroni correction and p-value <0.007 were considered statistical significant.

**Table 2:** Results from logistic regression test for association of rs2241423 to obesity in the Swedish cohort, adjusted for gender

**Swedish children**	**Genotype distribution AA/AG/GG (%)**	**MAF**	**HWE**	**Odds ratio** **(95% CI)**	**p-value**
All	52/359/564(5.3/36.8/57.8)		0.66		
Controls	33/199/282(6.4/38.7/54.9)	25.8% (256/519)	0.91	**0.79 (0.64 - 0.98)**	**0.029**
Obese	19/160/279(4.1/34.9/60.9)	21.5% (193/466)	0.58		

The minor allele of rs2241423 has a protective effect against obesity. MAF – minor allele frequency. HWE – result for test for deviation from Hardy Weinberg equilibrium, p-value. CI – confidence interval.

### Power analysis

Power calculations were carried out with the CaTS power calculator (http://www.sph.umich.edu/csg/abecasis/CaTS) [27] and QUANTO (http://hydra.usc.edu/gxe/). For the case/control comparison we had 80% power to detect an association with obesity with a relative risk of 1.34. For normalized quantitative phenotypes in the cohort of Greek children we had 80% power to detect an effect size (*β*) of 9.5% of the standard deviation per allele. For the cohort of Greek children of we had a 60% power to detect the observed effect size of *β* = −0.092. In the case–control study of Swedish obese children and matched controls, we had about 30% power to detect the observed OR of 0.79 (95% CI: 0.64–0.98).

## Results

### The minor allele of rs2241423 is associated with a protective effect against obesity in a cohort of Swedish children

The minor allele frequency of rs2241423 was 21.5% in the obese cohort and 25.8% in the control population. Logistic regression adjusted for gender showed the minor allele of rs2241423 to be associated with a protective effect against obesity (OR = 0.79, 95% CI = 0.64–0.98, *p* = 0.029) (Table 2). Linear regression analysis was unable to detect an association of rs2241423 with BMI within the obese children.

### The healthy growth study - the minor allele of rs2241423 is associated to a lower BMI

The minor allele frequency in the cohort of Greek children was 24.8%. Linear regression confirmed the association of the minor allele of rs2241423 with a lower BMI (*β*–0.09191, *p* = 0.028) observed in the Swedish child cohort (Table 3). The minor allele of rs2241423 was also associated with smaller hip circumference in the Greek children. A trend towards association of rs2241423 with other measurements of adiposity such as: tricep skinfold thickness, bicep skinfold thickness, subscapular skinfold thickness, suprailliac skinfold thickness and waist circumference was also observed (Additional file 2: Table S1). This was not observed when BMI was included as a covariate in the model. However, a trend towards an association of the minor allele rs2241423 with lower hip circumference was observed after including BMI as a covariate (*p* = 0.095) (Additional file 2: Table S1).

**Table 3:** Results from linear regression analysis of the association of rs2241423 to a higher BMI in the Greek Healthy Growth Study cohort, adjusted for gender and pubertal development (tanner stage)

**Greek children**	**Genotype distribution AA/AG/GG (%)**	**MAF**	**HWE**	**n**	***β***	**p-value**
rs2241423	138/865/1300(6.0/37.6/56.5)	24.8%	0.74	2282	−0.092	0.028

*β*- regression coefficient. MAF – minor allele frequency. HWE – result for test for deviation from Hardy Weinberg equilibrium, p-value.

### Linkage disequilibrium-analysis

Rs2241423 is located in a haplotype block of about 40 kb encompassing part of the last intron of MAP2K5 as well as a downstream region containing 10 kb (Additional file 1: Figure S1).This position is in close proximity to a number of potential regulatory sites according to the encyclopedia of DNA elements (ENCODE) (Figure 1).

## Discussion

We analyzed the association of rs2241423 to obesity and BMI in two cohorts of children from Sweden and Greece. The minor allele of rs2241423 was associated with a protective effect against obesity in the Swedish cohort, and lower BMI in the Greek cross-sectional cohort, which is in line with published observations on this locus in GWA-studies [1,2]. Analysis of an association of rs2241423 with measurements of body adiposity revealed an association of the minor allele with a smaller hip circumference in Greek children (Additional file 2: Table S1). However, this effect was not detectable when BMI was included as a covariate in the model suggesting that the effect of rs2241423 on body adiposity measurements is secondary to BMI. A trend towards an association of rs2241423 with lower hip circumference was however observed when including BMI as a covariate in the analysis (*p* = 0.095) (Additional file 2: Table S1). We were thus unable to positively report associations with novel phenotypes, perhaps due to the relatively small size of our cohort. Nevertheless, these results confirm the association presented by Speliotes et al. and demonstrates the effects of rs2241423 are detectable already at childhood.

Our results are consistent with previous meta-analyses by the GIANT consortium and Dorajoo et al. [1,2] considering the direction of the effect in cohorts with large age span. The GIANT consortium also reported subsequent meta-analyses focusing on children and revealed an association of rs2241423 with extreme obesity in children in case control studies. In the current study, we find an association to childhood BMI on a population level as well, further strengthening the association of this locus to early-onset overweight and obesity. Studies have suggested BMI to be more strongly influenced by environmental factors during childhood and adolescence, when the child is subjected to the eating habits of the family or environment it grows up in, compared to during adulthood when the child has gained its own independent eating habits [28].

The underlying functional mechanism of the association of rs2241423 variants with obesity, BMI and RLS is, as of yet, undetermined. The close proximity to transcription factor sites in the last exon of MAP2K5 may indicate an rs2241423-linked factor affecting transcription factor-binding and subsequent gene expression (Figure 1).

## Conclusion

In conclusion, results from the two child cohorts utilized in this study confirm the association of the minor allele of rs2241423 to a protective effect against obesity in children from the Swedish cohort, and with a lower BMI in a cross-sectional cohort of Greek children, which is in line with previous observations in GWAs [1,2]. This demonstrates the effects of rs2241423 to be detectable already at childhood.

## Competing interests

The author’s declare no conflict of interest.

## Authors’ contributions

MRA & JAJ administered the genotyping, conducted the data analysis and authored the draft. Management, collection of information and samples from the cohort of Swedish children was performed by AEE & CM. GM, YM & GPC managed, and collected information and samples from the cohort of Greek Children; JAJ, RF & HBS conceived and planned the study. All authors revised the manuscript and contributed to the discussion. All authors read and approved the final manuscript.

## Pre-publication history

The pre-publication history for this paper can be accessed here:

http://www.biomedcentral.com/1471-2350/13/36/prepub

## Supplementary Material

Additional file 1 **Figure S1. Linkage disequilibrium (LD) pattern of the proximal region of the obesity associated SNP rs2241423 (marked in red).** R-squared scores are used to visualize the LD pattern. Linkage disequilibrium-analysis using confidence intervals according to Gabriel et al. [28] identifies rs2241423 to lie in a haplotype block of about 40 kb encompassing the last intron and exon of MAP2K5 as well as a 10 kb downstream region. Click here for file

Additional file 2 **Table S1. Association of rs2241423 to measurements of body adiposity in greek children.** * - skewed quantitative variables were normalized by logarithmation. Click here for file
