# Supplementary material for: The MAP2K5-linked SNP rs2241423 is associated with BMI and obesity in two cohorts of Swedish and Greek children
Source: BMC Med Genet. 2012 May 17;13:36. doi: 10.1186/1471-2350-13-36 (PMC3459804; doi:10.1186/1471-2350-13-36)
Supplement: Additional file 2 — Table S1. Association of rs2241423 to measurements of body adiposity in greek children. * - skewed quantitative variables were normalized by logarithmation. [file 1471-2350-13-36-S2.docx]

**Figure Legends**

**Supplementary Figure 1.** Linkage disequilibrium (LD) pattern of the proximal region of the obesity associated SNP rs2241423 (marked in red). R-squared scores are used to visualize the LD pattern. Linkage disequilibrium-analysis using confidence intervals according to Gabriel et al. [27] identifies rs2241423 to lie in a haplotype block of about 40 kb encompassing the last intron and exon of MAP2K5 as well as a 10 kb downstream region.
